# Fabrication and Luminescence Properties of Highly Transparent Green-Emitting Ho:Y_2_O_3_ Ceramics for Laser Diode Lighting

**DOI:** 10.3390/ma17020402

**Published:** 2024-01-13

**Authors:** Yan Liu, Xianpeng Qin, Lin Gan, Guohong Zhou, Song Hu, Zhengjuan Wang, Juan Jiang, Tianjin Zhang, Hetuo Chen

**Affiliations:** 1 State Key Laboratory of High Performance Ceramics and Superfine Microstructure, Shanghai Institute of Ceramics, Chinese Academy of Sciences, Shanghai 200050, China; ly8267426@163.com (Y.L.); sic_zhough@mail.sic.ac.cn (G.Z.); yzhusong10@mail.sic.ac.cn (S.H.); wzhj926@mail.sic.ac.cn (Z.W.); chenhetuo@mail.sic.ac.cn (H.C.); 2 Collaborative Innovation Center for Advanced Organic Chemical Materials Co-Constructed by the Province and Ministry, Ministry of Education Key Laboratory for the Green Preparation and Application of Functional Materials, School of Material Science and Engineering, Hubei University, Wuhan 430062, China; jiangjuan@hubu.edu.cn (J.J.); zhangtj@hubu.edu.cn (T.Z.); 3 Center of Materials Science and Optoelectronics Engineering, University of Chinese Academy of Sciences, Beijing 100049, China; 4 Suzhou Research Institute, Shanghai Institute of Ceramics, Chinese Academy of Sciences, Taicang 215400, China

**Keywords:** highly transparent, Ho:Y_2_O_3_, green luminescent, laser lighting

## Abstract

Highly transparent Ho:Y_2_O_3_ ceramics for laser diode lighting were prepared using the vacuum sintering method with 0.3 at.% Nb_2_O_5_ as a sintering additive. The microstructures, transmittance, and luminescence properties of the Ho:Y_2_O_3_ ceramic samples were investigated in detail. The transmittance levels of all samples with various Ho^3+^ concentrations reached ~81.5% (2 mm thick) at 1100 nm. Under the excitation of 363 nm (ultraviolet) or 448 nm (blue) light, Ho:Y_2_O_3_ transparent ceramic samples showed that green emission peaked at 550 nm. The emission intensity was strongly affected by the concentration of Ho^3+^ ions, reaching its highest level in the sample doped with 1 at.% Ho^3+^. The CIE coordinates of the luminescence were in the green region (i.e., the CIE coordinates of the sample doped with 1 at.% Ho^3+^ were [0.27, 0.53] and [0.30, 0.69], under the excitation of 363 nm and 448 nm light, respectively). The possibility of its application as laser diode lighting was reported. Under the excitation of 450 nm blue laser, the sample doped with 0.5 at.% Ho^3+^ had the best performance: the saturated luminous flux, lumen efficiency, and the luminescence saturation power densities were 800 lm, 57.7 lm/W, and 17.6 W/mm^2^, respectively. Furthermore, the materials have high thermal conductivity and mechanical strength due to their host of rare-earth sesquioxide. Thus, Ho:Y_2_O_3_ transparent ceramics are expected to be a promising candidate for green-light-emitting devices for solid-state lighting, such as laser diode lighting.

## 1. Introduction 

In recent years, solid-state lighting has been widely studied due to its advantages of energy saving, high brightness, and long service life [1]. However, solid-state lighting devices based on LED or blue-laser diode (LD) have poor heat dissipation, which can easily cause aging problems. Seeking fluorescent host materials with high thermal conductivity is a major research direction, which is hoped to solve this problem of thermal management. Compared with phosphors, phosphors-in-glass, and single-crystal materials, phosphor transparent polycrystalline ceramics are considered to be an ideal candidate for phosphor converters due to their high thermal conductivity, heat resistance, and ease of production [2,3]. However, rare-earth (RE) ion doping is another major way to achieve transparent ceramic luminescence. Many studies have focused on the development of rare earth (RE)-based transparent polycrystalline materials [4]. It has been reported that sesquioxide materials (i.e., Y_2_O_3_, Lu_2_O_3_, Sc_2_O_3_) or garnet-based materials (i.e., Y_3_Al_5_O_12_, Lu_3_Al_5_O_12_) have similar ionic radii and chemical properties to rare-earth ions (REs) [5,6,7,8,9,10]. Therefore, these materials are good luminescent host materials. Y_2_O_3_, in particular, exhibits significant, advantageous optical and physical properties, such as a wide transparency range (230 to 8000 nm), lower phonon energy levels (i.e., 565 cm^−1^), phase permanency, higher thermal conductivity (i.e., ~17 W/(m·K)), etc. [11]. Therefore, transparent polycrystalline Y_2_O_3_ is widely considered as a potential host material thatcan be used in high-power laser illumination, such as deep-sea lighting, automotive laser headlights, and blue-laser diode (LD).

Rare-earth ions (REs) are widely used as luminescent centers in polycrystalline materials such as Nd^3+^, Er^3+^, Ho^3+^, Yb^3+^, Tm^3+^, etc. Rare earth (RE)-based transparent polycrystalline materials have applications in lasers, solid-state lighting, etc. Among the rare-earth ions are Ho^3+^ ions, which are used for laser action at different wavelengths, from 550 to 3900 nm. For example, Ho^3+^-ion-doped polycrystalline transparent ceramics have been widely studied in 2000 nm solid-state lasers due to their ^5^I_7_ → ^5^I_8_ energy-level transitions [12,13,14]. The Ho^3+^ ion is also a suitable active ion for the up-conversion laser and many researchers have focused on up-conversion emissions (for example, highly efficient up-conversion luminescence in a Ho^3+^–Yb^3+^ system) [15]. Furthermore, under the excitation of ultraviolet and visible light, Ho^3+^ ions produce a high-intensity green light emission, especially in the green region, and can, therefore, be used as an element in white light production due to their green emission. In addition, in dark conditions, human vision is the most sensitive to green light. Therefore, green-luminescent materials play an important role in various color converters. Lu_3_Al_5_O_12_:Ce (LuAG:Ce) is a well-known green-color-changing material with high thermal stability and high efficiency. However, its wide emission spectrum and the price of raw Lu materials limit its application [16]. YAGG:Ce^3+^ is also a promising green-luminescent material, but the controllable preparation of YAGG:Ce ceramics is still a challenge due to the volatilization of Ga at high temperatures [17]. According to previous studies, Ho:Y_2_O_3_ phosphors can be applied to display applications and light-emitting diodes for green-production phosphors due to effective green luminescence [18]. Yet, at present, there are few studies that have focused on their role in green-laser lighting in Y_2_O_3_ polycrystalline transparent ceramics.

Owing to the refractoriness of yttria (melting point: 2430 °C) [19], it is difficult to fabricate highly transparent yttria ceramics. For transparent ceramic light functionalization applications, the premise is to ensure that the ceramics have a high transmittance. In our previous work, Nb_2_O_5_ was proven to be an efficient sintering additive for Y_2_O_3_ transparent ceramics [20]. When Nb_2_O_5_ is used as the sintering additive of Y_2_O_3_, the thermal conductivity of ceramics is close to the theoretical thermal conductivity level due to its small doping amount (~0.3 at.%), which is beneficial for the thermal management and high-power luminescence of ceramics. Luminescent materials with a high thermal conductivity and high optical transparency are also very important and there is justification to explore the possible application of Y_2_O_3_ polycrystalline transparent ceramics in the field of green luminescence. In this work, highly transparent green-emitting Ho:Y_2_O_3_ ceramics with various Ho concentrations were fabricated for laser-diode lighting using the vacuum sintering method, with Nb_2_O_5_ as a sintering additive. The phase composition, microstructures, transmittance, and luminescence properties of the Ho:Y_2_O_3_ ceramic samples were investigated. The results demonstrate that the Ho:Y_2_O_3_ ceramic samples had a high transmittance level and good green luminescence properties, which make them suitable green candidate materials for laser lighting.

## 2. Experimental Procedure

Commercially available Y_2_O_3_ (99.99%, Jiangyin Jiahua, China), Ho_2_O_3_ (99.995%, Rare-Chem, Huizhou, China), and Nb_2_O_5_ (99.99%, Macklin, Huizhou, China) powders were used as starting materials. According to the compositions of (Y_(1−x)_Ho_x_)_2_O_3_ (x = 0, 0.005, 0.01, 0.02, and 0.03), the concentrations of Ho_2_O_3_ were 0, 0.5, 1, 2, and 3 at.%, respectively. The samples were designated as Ho0, Ho0.5, Ho1, Ho2, and Ho3, respectively, and the powders were weighed and milled through planetary milling with 0.3 at.% Nb_2_O_5_ as the sintering additive, using 3 mm ZrO_2_ balls in anhydrous ethanol at 250 rpm for 24 h. After that, the slurries were dried at 55 °C for 24 h, and then the powders were sieved through a 200-mesh screen and were subsequently calcined at 1000 °C for 4 h to remove all organic components completely. The calcined powders were pressed into Φ20 mm pellets at a pressure of 7.5 MPa in a stainless-steel mold, and then they were subjected to cold isostatic pressing (CIP) treatment at 200 MPa. Then, the pellets were calcined at 1200 ℃ for 2 h to enhance the strength of the green. The pre-sintered samples were sintered in a vacuum furnace at 1780 °C for 14 h under a vacuum of ~10^−3^ Pa. The sintered samples were then annealed in air at 1400 °C for 5 h. Finally, all the sintered samples were double-side polished to a thickness of 2 mm for measurements.

The crystalline phases were carried out using X-ray diffraction (XRD, D/max 2550V, JPAT, Tokyo, Japan) analysis with Cu Kα radiation (λ = 1.5406 Å). The lattice parameter of the ceramic samples was calculated according to the XRD results. The unit-cell volume and theoretical density were calculated according to the lattice parameters using Equation (1) [21],

*ρ_th_* = *ZM*/*NV* (1)

where *Z* is the molecule number in a unit cell (*Z* = 16), *M* is the molecular weight, *N* is Avogadro’s constant, and *V* is the unit-cell volume. The bulk densities of the Ho:Y_2_O_3_ ceramics were measured using the Archimedes method. Finally, the relative density of a ceramic sample was calculated. The microstructures of the samples were detected using a scanning electron microscope (SEM, TM-3000, HITACHI, Tokyo, Japan). Optical transmittance and absorbance spectra of the samples were measured using a UV–VIS–NIR (V770, JASCO, Tokyo, Japan) spectrometer in the range from 190 to 1200 nm. Fluorescence spectra were measured using low-temperature fluorescence spectrometry (FLS920, Edinburgh Instruments, Edinburgh, UK). The luminescence properties of Ho:Y_2_O_3_ ceramics under different incident laser powers were measured in the reflection mode using an integrating sphere (diameter 30 cm, Labsphere Inc., Sutton, NH, USA), CCD spectrometer (OHSP-350 M, Hopoocolor Technology Co., Ltd., Hangzhou, China), 450 nm blue laser (LSR-PS-FA, Lasever, Ningbo, China), high-power light source, and incident power controller. The laser spot diameter was adjusted to 1 mm.

## 3. Results and Discussion

**Figure 1:**
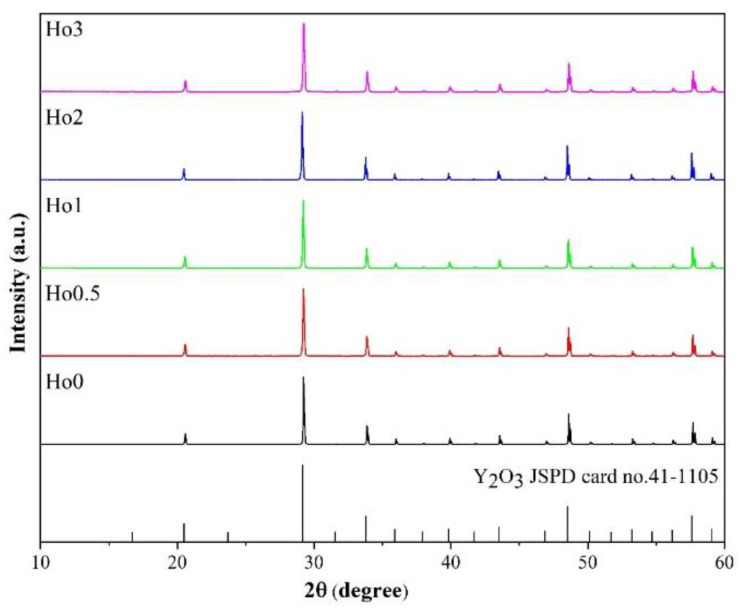
XRD patterns of the Ho:Y_2_O_3_ ceramics doped with various Ho concentrations.

Figure 1 shows the XRD patterns of the ceramic samples sintered at 1780 °C for 14 h, doped with various Ho concentrations. It was confirmed that all samples were composed of a single cubic-yttria phase without impurity phases. All the detected diffraction peaks corresponded to the standard card (JSPD card no. 41-1105). According to previous studies, a small amount of Nb^5+^ could be incorporated into the lattice of Y_2_O_3_ at high temperatures [20]. In this study, the XRD results imply that the Nb^5+^ and Ho^3+^ ions were incorporated into the yttria lattice. Table 1 tabulates the lattice parameters, unit-cell volumes, theoretical densities (*ρ_th_*), and relative densities (*ρ_re_*) of the ceramic samples, which were calculated using the XRD results. The lattice parameters and cell volume of Ho:Y_2_O_3_ ceramics demonstrated minimal change with the doping of Ho ions, owing to the Ho^3+^ (0.894 Å, CN6) radius [22], which is similar to that of Y^3+^ (0.900 Å, CN6). Because the atomic weight of Ho is larger than that of Y, as Ho^3+^-ion doping concentration was increased, the theoretical density of the ceramic samples increased gradually. However, the relative density of the ceramic samples hardly changed, regardless of the doping concentration, with all of the samples having a dense structure (i.e., reaching more than 99.9%). The uniform single phase and dense structure ensured the high transmittance level of the ceramics.

**Figure 2:**
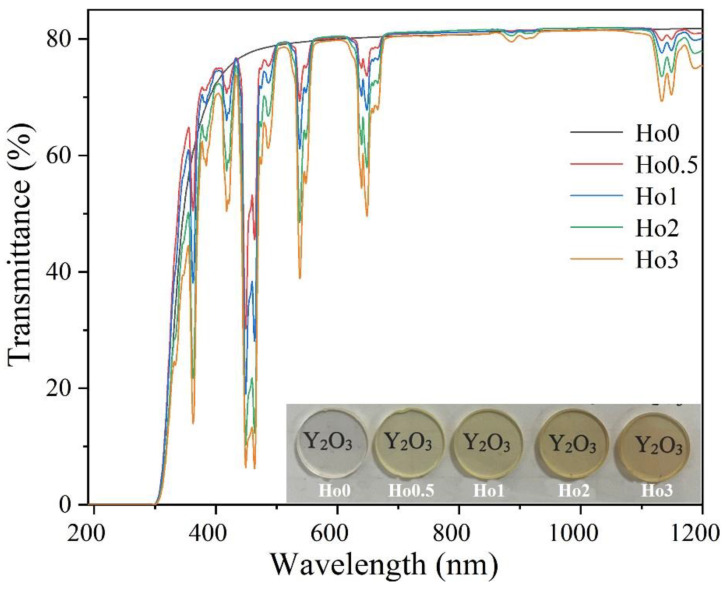
In-line transmittance levels of the samples doped with various Ho^3+^ concentrations (2 mm thick); the insert shows the digital photographs of the samples.

The illustration in Figure 2 displays photographs of the Ho:Y_2_O_3_ ceramics doped with various Ho concentrations. It is evident that the samples were highly transparent, as the words below them can be clearly seen. With the increase in the Ho^3+^ concentration, the color of the samples gradually deepened. The in-line transmittances of the samples doped with various Ho^3+^ concentrations are shown in Figure 2. All samples showed high transmittance levels; the transmittance rates at the wavelengths of 400 and 1100 nm were ~74% and ~81.5%, respectively. The transmittance level was close to the theoretical transmittance. Notably, the sample had a high transmittance in the visible light range, guaranteeing that there was no apparent light absorption for the green light emitted by the ceramics. According to our previously published literature, the transmittance of the samples is comparable to the optimum transmittance of Y_2_O_3_ ceramics sintered with Nb_2_O_5_ as the sintering additive [20]. The incorporation of Ho^3+^ ions had no significant effect on the transmittance of the ceramic samples. When Ho^3+^ ions were doped into an Y_2_O_3_ lattice, the samples had strong absorption in the visible light band (i.e., at the wavelengths of 363, 420, 448, 538, and 647 nm), and the absorption of these bands gradually increased with the increase in Ho^3+^ concentrations. The absorption peak in the transmittance curve is consistent with that when Zr^4+^ is used as the sintering additive [13]. The absorption of ceramics in these bands led the ceramics to show a certain color, which is consistent with the physical picture (see Figure 2). Table 2 lists the average wavelengths of Ho^3+^-ion transitions in yttria transparent ceramics [18,23], which correspond to the absorption peak in the transmittance curve.

**Figure 3:**
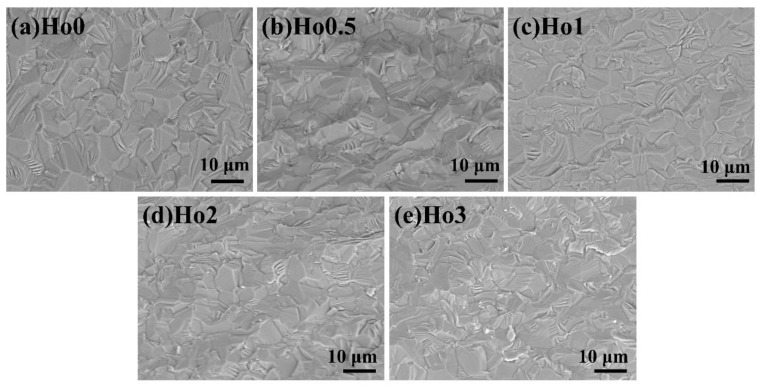
SEM images of the samples with the following Ho concentrations: (**a**) Ho0 (**b**) Ho0.5, (**c**) Ho1, (**d**) Ho2, and (**e**) Ho3.

Figure 3a–e illustrate the SEM images of the fracture surfaces for samples with various Ho^3+^ concentrations. All samples showed dense microstructures, and no apparent residual pores were observed, which is consistent with the results of high relative density (see Table 1). Furthermore, no obvious second phase was observed in the interior of the ceramic or at the grain boundary. The fracture mode of ceramics is the coexistence of transgranular fracture and intergranular fracture. At the same time, it was found that the grain size of the ceramics hardly changed with various Ho concentrations. Doping with rare-earth Ho^3+^ had little effect on the microstructure of the ceramics. The grain size of all samples was ~6.5 μm. The above results are consistent with our previous work, which demonstrated that Nb_2_O_5_ is an effective sintering additive of Y_2_O_3_ transparent ceramics and can result in a denser microstructure in the ceramic samples [20]. This ensures that the ceramic sample has good mechanical properties, so that it may be suitable for application to laser-diode lighting.

**Figure 4:**
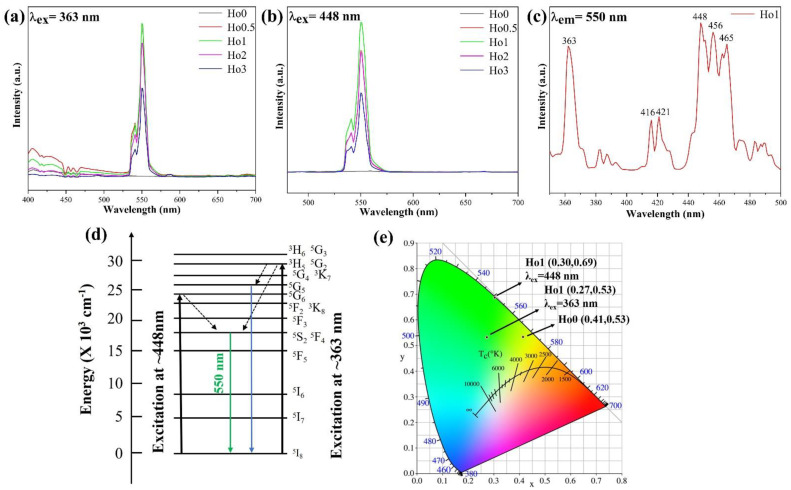
(**a**) and (**b**) PL emission spectra of Ho:Y_2_O_3_ transparent ceramics ((**a**) λ_ex_ = 363 nm, and (**b**) λ_ex_ = 448 nm). (**c**) PL excitation spectrum of 1 at.% Ho:Y_2_O_3_ transparent ceramic (at 550 nm). (**d**) Energy-level diagram of the Ho^3+^ ion. (**e**) The CIE coordinates of 1 at.% Ho:Y_2_O_3_ transparent ceramic on a 1931 CIE chromaticity diagram (in the green region).

It can be seen in the transmittance curve that the Ho:Y_2_O_3_ transparent ceramic samples had strong absorption peaks at 363 nm and 448 nm (see Figure 2). To study the luminescence properties, the room temperature photoluminescence (PL) spectra of the samples were measured under 363 nm and 448 nm. Figure 4a,b show the PL emission spectra of the Ho:Y_2_O_3_ transparent ceramics. Under the excitation of 363 nm (ultraviolet) and 448 nm (blue) light sources, the Ho:Y_2_O_3_ transparent ceramic samples doped with Ho^3+^ ions emitted green light centered at 550 nm (green, ^5^S_2_, ^5^F_4_ → ^5^I_8_) [18,24], and the green light emission intensity was affected by the concentration of Ho^3+^ ions: the green fluorescence intensity increased first and then decreased with an increase in the doping concentration. The material formed well-crystallized, micron-sized grains, which led the ceramic sample to emit only from two excited states (^5^F_4_ and ^5^S_2_) and did not cause the emission of ^5^F_5_ → ^5^I_8_ (645 nm). The closely lying, less intense peaks in the emission spectra are due to the splitting of the Stark levels of ^5^S_2_, and ^5^F_4_ levels of Ho^3+^ ion, due to the crystal field of Y_2_O_3_ [18]. When doping with 1 at.% Ho^3+^, the green fluorescence intensity reached its maximum. The decrease in fluorescence intensity with the increase in the doping concentration is mainly due to the concentration quenching effect of the emission center. The variation in green luminescence intensity with Ho^3+^ concentration was consistent with the results reported by Singh et al. [18]. Contrary to findings following excitation with a 448 nm light source, when excited at 363 nm, the ceramic samples also had a weak emission peak in the wavelength range of 400–475 nm (^5^G_5_ → ^5^I_8_) [25], and the emission peak intensity was much lower than that of green light emission.

The luminescence process occurs as follows. When excited by a 363 nm light source, a green and blue emission is generated through an excitation process (Figure 4d):

^5^I_8_ → ^3^H_5_/^5^G_2_ (ground state absorption).
^3^H_5_/^5^G_2_ → ^5^G_5_ or ^5^S_2_/^5^F_4_ (multiphonon relaxation).
^5^G_5_ or ^5^S_2_/^5^F_4_ → ^4^I_15/2_ (radiative relaxation).

When excited by a 448 nm light source, a green emission is generated through a process including ground state absorption and multiphonon relaxation:

^5^I_8_ → ^5^G_6_ (ground state absorption).
^5^G_6_ → ^5^S_2_, ^5^F_4_ (multiphonon relaxation).
^5^S_2_, ^5^F_4_ → ^4^I_15/2_ (radiative relaxation).

The excitation spectrum for the Ho:Y_2_O_3_ transparent ceramics, corresponding to the emission at ~550 nm (in the 350–500 nm region), is shown in Figure 4c. The PL excitation spectrum has several narrow peaks in the 350–500 nm region. The narrow peak is due to the f-f transition in Ho^3+^ ions, and the strongest peak is at 448 nm. The narrow peaks at ~363, ~420, ~448, and ~460 nm are due to the energy-level transitions ^5^I_8_ → ^5^G_2_, ^3^H_5_/^5^I_8_ → ^5^G_5_, ^5^I_8_ → ^5^G_6_, and ^5^I_8_ → ^5^F^2^/^3^K_8_ of Ho^3+^ ions, respectively [24,26].

The color purity of a substance can be recognized in terms of color coordinates, known as Commission International De I’Eclairage (CIE) chromaticity coordinates. The CIE chromaticity diagrams for the Ho:Y_2_O_3_ transparent ceramics excited at 363 and 448 nm are shown in Figure 4e. Under the excitation of 363 nm (ultraviolet) and 448 nm (blue) light sources, the CIE coordinates were (0.27, 0.53) and (0.30, 0.69), respectively. The difference in the CIE coordinates is due to the fact that the ceramic samples had a lower emission in the blue band under the excitation of 363 nm; therefore, the color coordinates of the samples have a tendency to move toward the blue region compared to 448 nm. Compared with the LuAG:Ce or YAGG:Ce system (a broad emission band with an FWHM of approximately 100 nm) [16,17], the green emission peak of the Ho:Y_2_O_3_ ceramic sample was narrower, and the FWHM of the emission peak was smaller. The PL spectra of the ceramics show a narrow emission band centered at 550 nm, with an FWHM of approximately 12 nm under 363 or 448 nm excitation. The Ho:Y_2_O_3_ transparent ceramics show good monochromaticity. Therefore, under the excitation of 363 nm (ultraviolet) and 448 nm (blue) light sources, the color coordinates are located in the green region (see Figure 4e). These results show that the Ho:Y_2_O_3_ ceramics can be excited by different wavelength light sources to meet the needs of green luminescence.

**Figure 5:**
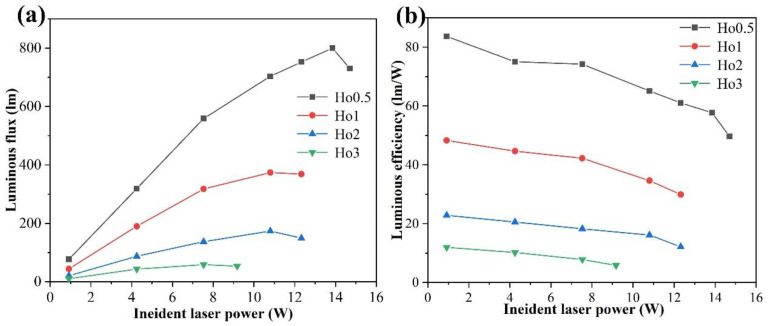
The intensity of (**a**) luminous flux and (**b**) luminous efficiency for different samples (2 mm thick) under various input blue laser power densities.

In order to explore the possible applications of Ho:Y_2_O_3_ ceramics in the field of laser-diode lighting, we investigated the performance of Ho:Y_2_O_3_ ceramics excited by the 450 nm blue laser and the optical properties of Ho:Y_2_O_3_ ceramics with different doping Ho^3+^ concentrations and at different powers. All samples had a thickness of 2 mm. The luminous flux of all samples increased with the increase in excitation power, and then decreased after reaching luminescence saturation. With the increase in Ho concentration, the luminous flux of the sample decreased gradually under the same excitation power and the luminescence saturation power density dropped from 17.6 to 9.6 W/mm^2^ (the diameter of the laser spot was 1 mm). In yttria transparent ceramics, the incorporation of luminescent ions reduces the thermal conductivity of ceramics [27]. With the increase in Ho^3+^ doping concentration, the thermal conductivity of the Ho:Y_2_O_3_ ceramic samples gradually decreases [28]. Because the thermal stability that determines the saturation gradually decreases with the increase in the doping ion concentration, the Ho0.5 sample exhibited better luminescence properties, considering its saturated luminous flux and lumen efficiency can reach 800 lm and 57.7 lm/W, respectively (see Figure 5a,b). When the Ho^3+^ doping concentration increases gradually, an abnormal phenomenon appears and both luminous flux and lumen efficiency decrease, due to the thermal quenching effect. This can be explained by the fact that the higher concentration of Ho^3+^ content will directly lead to the production of more heat and, thus, the rapid increase in temperature will damage the color conversion [29]. According to the room temperature PL spectrum of the sample (see Figure 4a,b), it can be seen that the luminescence intensity is the largest when the Ho^3+^ doping concentration is 1.at %, and the gradual decrease in the luminescence intensity with the increase in the later concentration is due to concentration quenching. Most of the energy loss in the process of laser light conversion is radiated out in the form of heat, which will cause the temperature of the ceramics to rise rapidly. However, when testing the laser performance, there was no heat dissipation assembly of the ceramics, in which the heat conduction effect would be reduced, and the temperature would have a great influence on the luminescence performance of the ceramic sample (i.e., the thermal quenching of the luminescence center). In this experiment, the relationship between the luminous flux and the concentration of the sample was affected by the concentration and temperature (i.e., the concentration quenching and thermal quenching), and temperature was the main influencing factor in the test. Therefore, in order to obtain excellent lighting performance, it is necessary to enhance the thermal stability of the ceramics and enhance the heat dissipation performance of the device. For instance, adding a metal radiator, or a high-thermal-conductivity substrate to color converters, are two potential methods.

Ceramics can maintain the spectral and thermal properties required for single crystals; therefore, ceramic materials can be used effectively as laser host materials. Compared with single-crystal materials, Ho:Y_2_O_3_ transparent ceramic materials have the advantages of a lower preparation temperature and mass production [30]. For green luminescence properties, compared with the currently reported YAGG:Ce phospher-in-glass (i.e., with an upper limit of luminous flux and saturated power density of ~551 lm and 2.77 W/mm^2^, respectively) [17], Ho:Y_2_O_3_ ceramic samples have a higher luminous flux upper limit and saturated power density. Due to the high thermal conductivity of yttria ceramics compared to glass materials, yttria ceramics can better dissipate the accumulated heat during laser irradiation, resulting in better laser performance and the ability to withstand higher power input. Therefore, Ho:Y_2_O_3_ transparent ceramics may serve as a promising green color converter for laser-driven lighting.

## 4. Conclusions

For the first time, highly transparent green-emitting Ho:Y_2_O_3_ ceramics for laser-diode lighting were prepared using the vacuum sintering method with 0.3 at.% Nb_2_O_5_ as a sintering additive. The transmittance of all samples at 1100 nm reached ~81.5% (2 mm thick), which was close to the theoretical transmittance. The samples emitted green light centered at 550 nm under the excitation of both 363 nm (ultraviolet) and 448 nm (blue) light sources. Moreover, the luminescence intensity of the samples was significantly affected by the concentration of Ho^3+^ ions and reached its maximum when the doping concentration of Ho^3+^ ions was 1 at.%. Under the excitation of 450 nm blue laser, the saturated laser-power density of the sample decreased from 17.6 to 9.6 W/mm^2^ with an increase in the Ho^3+^ doping concentration. When doping with 0.5 at.% Ho^3+^, the sample had the best performance, with the saturated luminous flux and lumen efficiency at 800 lm and 57.7 lm/W, respectively. Overall, this study has shown that Ho:Y_2_O_3_ transparent ceramic is a green fluorescent material with considerable luminescence properties, which has the potential to be used for green-light-emitting devices for solid-state lighting such as laser-diode lighting.

## Figures and Tables

**Table 1:** Lattice parameters, unit-cell volumes, theoretical densities, and relative densities of the samples doped with various Ho^3+^ concentrations.

Samples (at.%)	Lattice Parameters *a* = *b* = *c* (nm)	Unit-Cell Volume (nm^3^)	Calculated Theoretical Density (g/cm^3^)	Relative Density (%)
Ho0	1.0604	1.1924	5.0335	≥99.9
Ho0.5	1.0604	1.1924	5.0505	≥99.9
Ho1	1.0603	1.1920	5.0674	≥99.9
Ho2	1.0603	1.1920	5.1027	≥99.9
Ho3	1.0602	1.1917	5.1366	≥99.9

**Table 2:** The mean wavelength for several Ho^3+^ transitions in Y_2_O_3_ transparent ceramics.

Transition from ^5^I_8_	Λ (nm)
^5^I_6_	1152
^5^I_5_	925
^5^F_5_	647
^5^S_2_,^5^F_4_	538
^2^F_2_,^3^K_8_,^5^G_6_,^5^F_1_	448
(^5^G,^2^G)_5_	420
(^5^G,^3^H)_5_,^3^H_6_, (^5^F,^3^F,^5^G)_2_	363

## Data Availability

Data are contained within the article.
